# CONTINUING BONDS OR UNBREAKABLE VOWS?: CONJUGALLY BEREAVED WOMEN'S PERCEPTIONS ABOUT REPARTNERING

**DOI:** 10.1093/geroni/igac059.244

**Published:** 2022-12-20

**Authors:** Sara Hackett

**Affiliations:** University of South Florida, Tampa, Florida, United States

## Abstract

The death of a romantic partner is regarded as one of life's most traumatic events. Despite established benefits from repartnering, women are typically less likely to repartner than men. Women oftentimes live longer than men and carry their grief into old age, but little is known about their integration of their loss into their lives going forward. Twenty conjugally bereaved women (Mean age = 78) participated in one 90-minute semi-structured interview to discuss their grief and their perceptions of their futures without their deceased partner. Thematic analysis revealed that participants viewed the deceased as an integral member of their present-day social convoy. Furthermore, participants expressed that they were averse to entering new romantic relationships because of either allegiance to the deceased, hesitancy about their age, or disinterest in caregiving for a subsequent partner. By exploring the reasons behind women’s resistance to repartnering, interventions may be developed to better support them.

